# Supplementary material for: Mechanical impact of epithelial−mesenchymal transition on epithelial morphogenesis in Drosophila
Source: Nat Commun. 2019 Jul 4;10:2951. doi: 10.1038/s41467-019-10720-0 (PMC6609679; doi:10.1038/s41467-019-10720-0)
Supplement: Supplementary file 1 — Supplementary Information [file 41467_2019_10720_MOESM1_ESM.pdf]

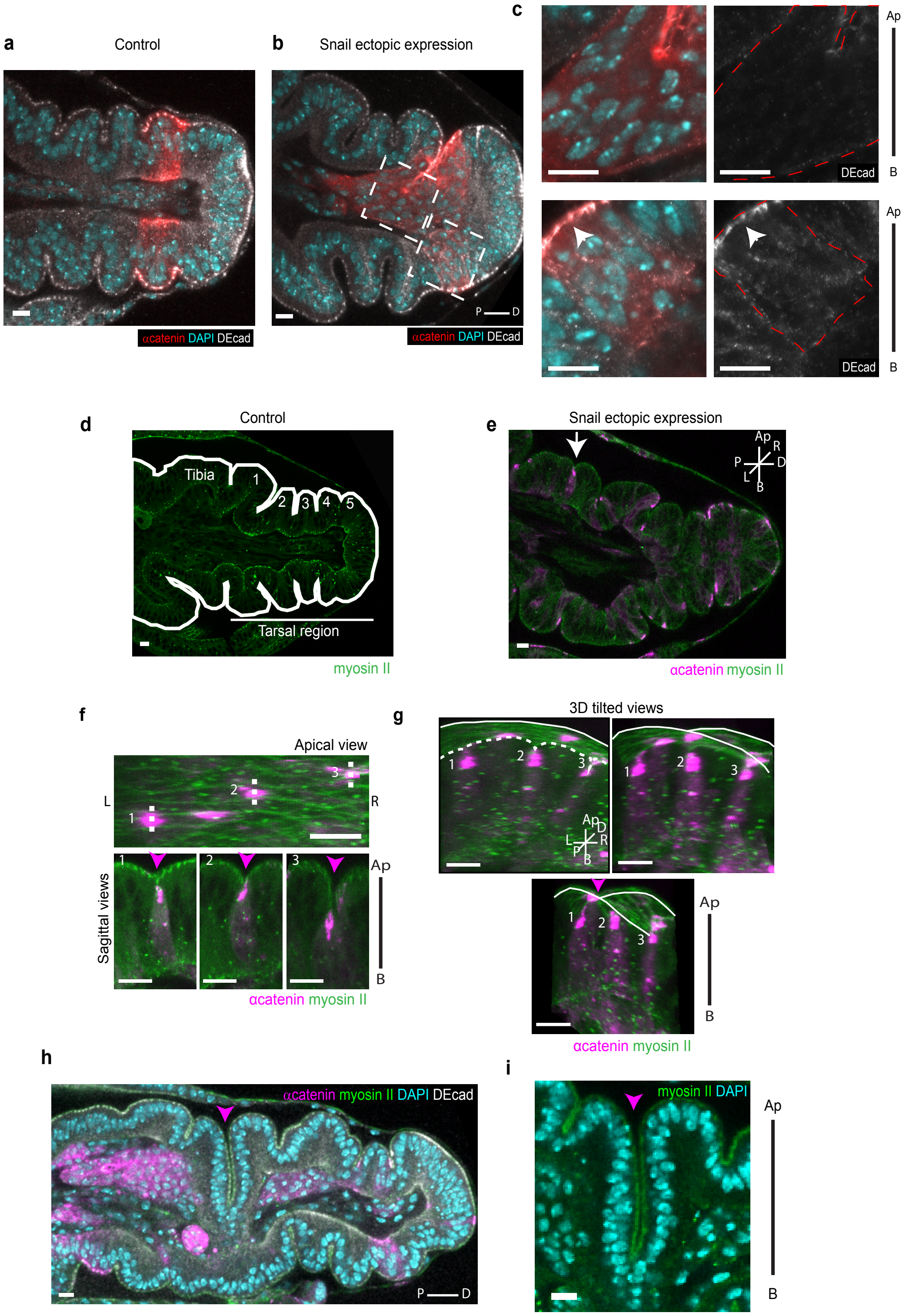

Supplementary Figure 1. EMT participates actively in tissue folding (Related to Figure 1 and 2).
a-c. Sagittal view of a leg disc expressing Snail in the apterous domain (b-c, ap-Gal4; UAS-Snail, n=13) or not (a). General view is on the left, close-up are on the right corresponding to the dotted squares in b. DE-cadherin is in white. Note the absence of DE-cadherin in cells that have delaminated (top close-up), while it is present in cells that are still integrated in the epithelium (bottom close-up). Scale bar: 10µm
d-e. Sagittal views of leg discs with (e) or without (d) Snail ectopic clones (marked in magenta by α-catenin-RFP expression in a sqh::sqh::GFP context). Normal folds from the tibia to the T5 tarsal segment are outlined in white in the control leg. An ectopic fold is formed in the tibia area in between three Snail expressing clones. Only one of them is visible in the z section shown in e (white arrow). Scale bar: 10µm.
f. Apical view (top) and sagittal sections (bottom) of three Snail-expressing clones (1, 2 and 3; the clone 2 is shown in e). The dotted lines on the apical view indicate the section planes of the sagittal views presented below. Each clone produces a deformation of different depth (magenta arrowheads). Scale bar: 10µm.
g. Different tilted 3D projections of the ectopic fold (magenta arrow) showing that the fold has formed in between the three clones. The apical surface is outlined in white at the border of the tissue and in dashed white along the fold. Scale bar: 10µm. See also Supplementary Movie 3.
h-i. Sagittal view of a leg disc with Snail expressing clones (marked in magenta by α-catenin::RFP expression). Note the presence of delaminated Snail expressing cells below the epithelium and the presence of an abnormally deep fold (magenta arrowhead, close-up in i). Compare the abnormal fold in h (magenta arrowhead) with folds in the control (d). Scale bar: 10µm.

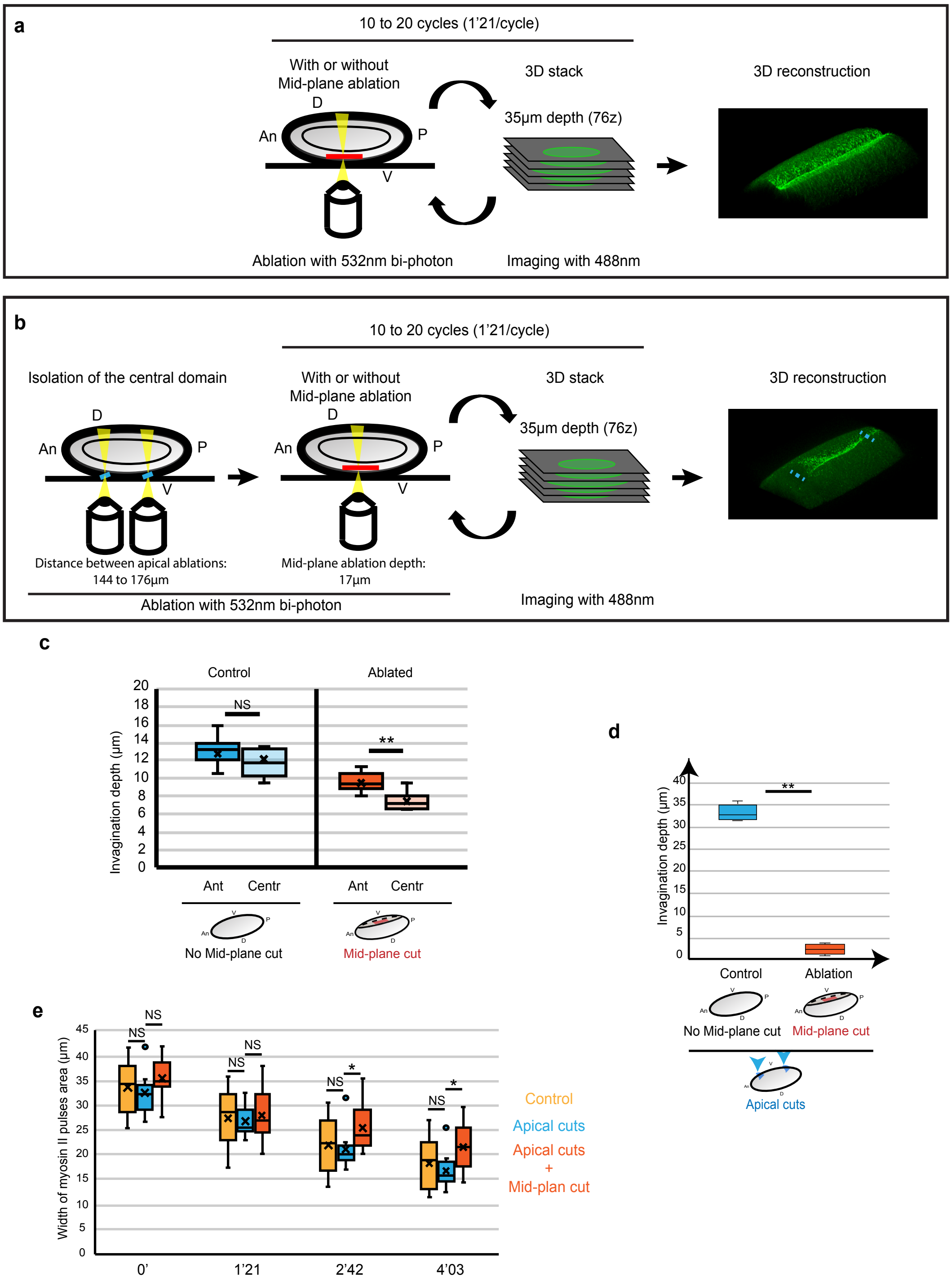

Supplementary Figure 2. Laser ablation set-up and quantification of the invagination depth (Related to figure 6).
a. Midplane ablation set-up: Ablation is performed with a 532nm pulsed laser, through the ventral side of the embryo, in one particular plane located at 17µm deep (cell mid-plane, red line), then a z stack is acquired with the 488nm laser. 10 to 20 cycles of ablation/z imaging are performed, with a time interval of 1’21. Morphogenesis defects are then analyzed on 3D reconstruction images.
b. Isolation of the central domain + midplane ablation set-up: Ablations are performed on the apical surface of the embryo, following a line (blue line) to isolated the central domain of the mesoderm. Then 10 to 20 cycles of midplane ablations/Z stack imaging are performed, with a time interval of 1’21. Morphogenesis defects are then analyzed on 3D reconstruction images.
c. Relative invagination depth of the central region (Centr) compared to the anterior (Ant) region at t=8’06 in control (Ctl) and Ablated embryos (see Methods). Statistical significance was assessed by the Wilcoxon signed-rank test (Ctl: n=10 embryos, p=0.17; Ablated: n=10, p=0.0024 – significant **).
d. Measure of invagination depth at the end of invagination process after isolation of the central region (two apical cuts) alone (Control) or in association with mid-plane cut (Ablation). Statistical significance was assessed by the Mann-Whitney test between Control and Ablation contexts (Ctl: n=5 and Ablated: n=7; p=0.0013 – significant **).
e. Quantification of the width of apical myosin pulses region in control (orange), after apical cuts (blue) and after apical cuts and mid-plane ablations (red) at different time points at the invagination stage. For each time point, statistical significance was assessed by the Mann-Whitney test between Control and Apical cuts contexts and between Apical cuts and Apical cuts + Mid-plan cut (Control: n=21, Apical cuts: n=10 and Apical cuts + Mid-plan cut: n=10; p<0.05 – significant *; p>0.05 - None Significant NS).
t0 has been defined as described in Methods.
An: anterior, P: posterior, D:dorsal, V:ventral

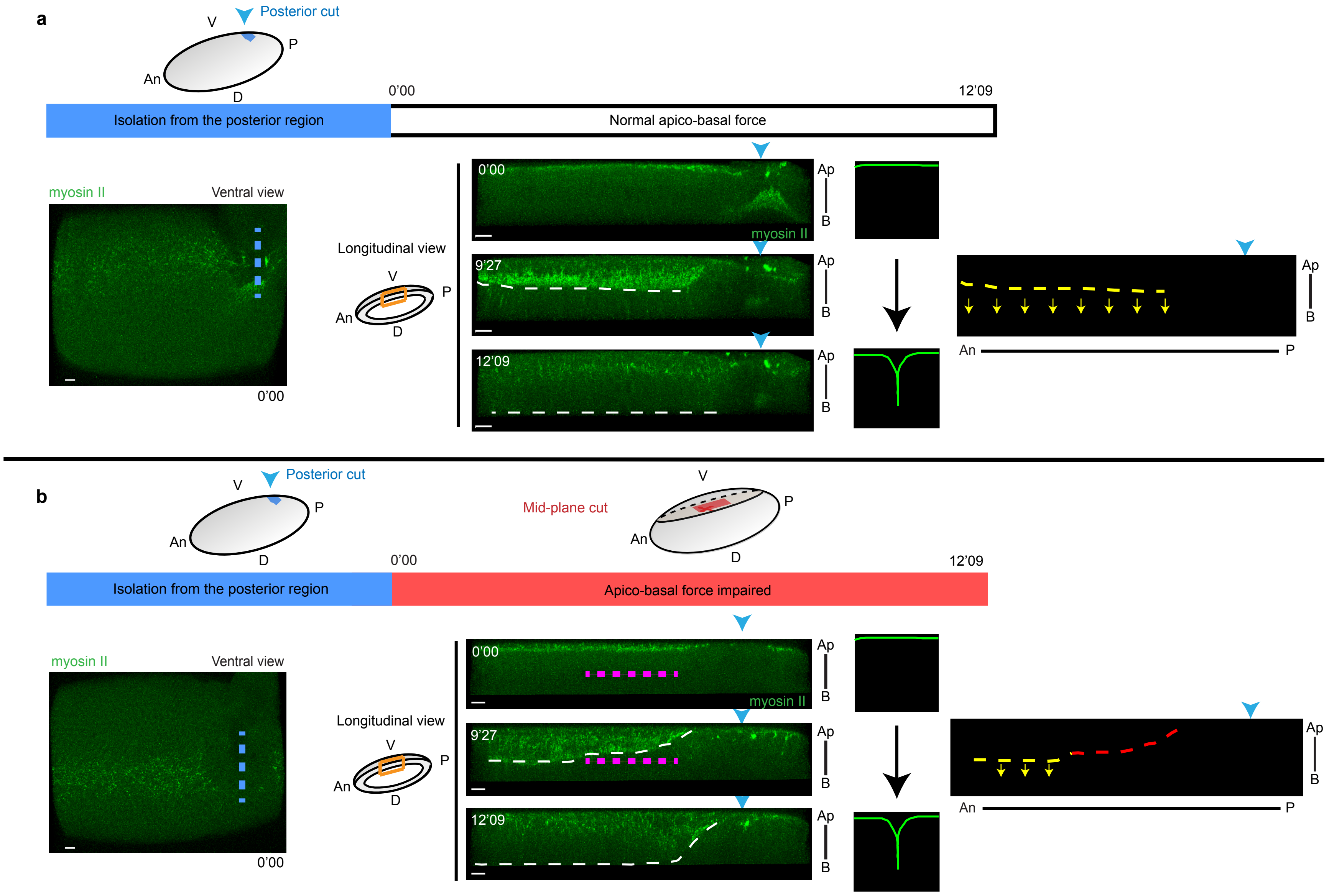

Supplementary Figure 3. An apico-basal force drive mesoderm invagination: Apico-basal forces generated in part of the mesoderm are sufficient to drag the invagination of the neighbouring tissue (Related to Figure 6).

a, b. Schemes recapitulate the laser cuts carried out in this set of experiments. First, the apical surface was cut on the posterior side (blue lines, posterior cut); then, apico-basal ablation was performed in the mid-plane of the central region of the mesoderm (b, red rectangle or dashed magenta line, mid-plane cuts) or not (a). Left: sqhKI[eGFP] embryo (ventral view) after apical cuts (dashed blue lines). Note the recoil induced by each cut. Center: time-lapse images of mesoderm invagination (longitudinal section). Schemes of the transversal sections with an outline of the apical surface in green are presented on the right.
Blue arrowheads denote the apical posterior cut and the white dashed line outlines the invagination front. Note that the invagination front is straight in the control condition (a, n=12), whereas it is curved and asymmetric when apico-basal forces are disrupted in the central region (b, n=5). Far right: outline of the invagination front line (longitudinal view) for each experiment, showing the delay in the invagination of the central region (red dashed line). Yellow arrows represent apico-basal forces.
Scale bars: 10µm

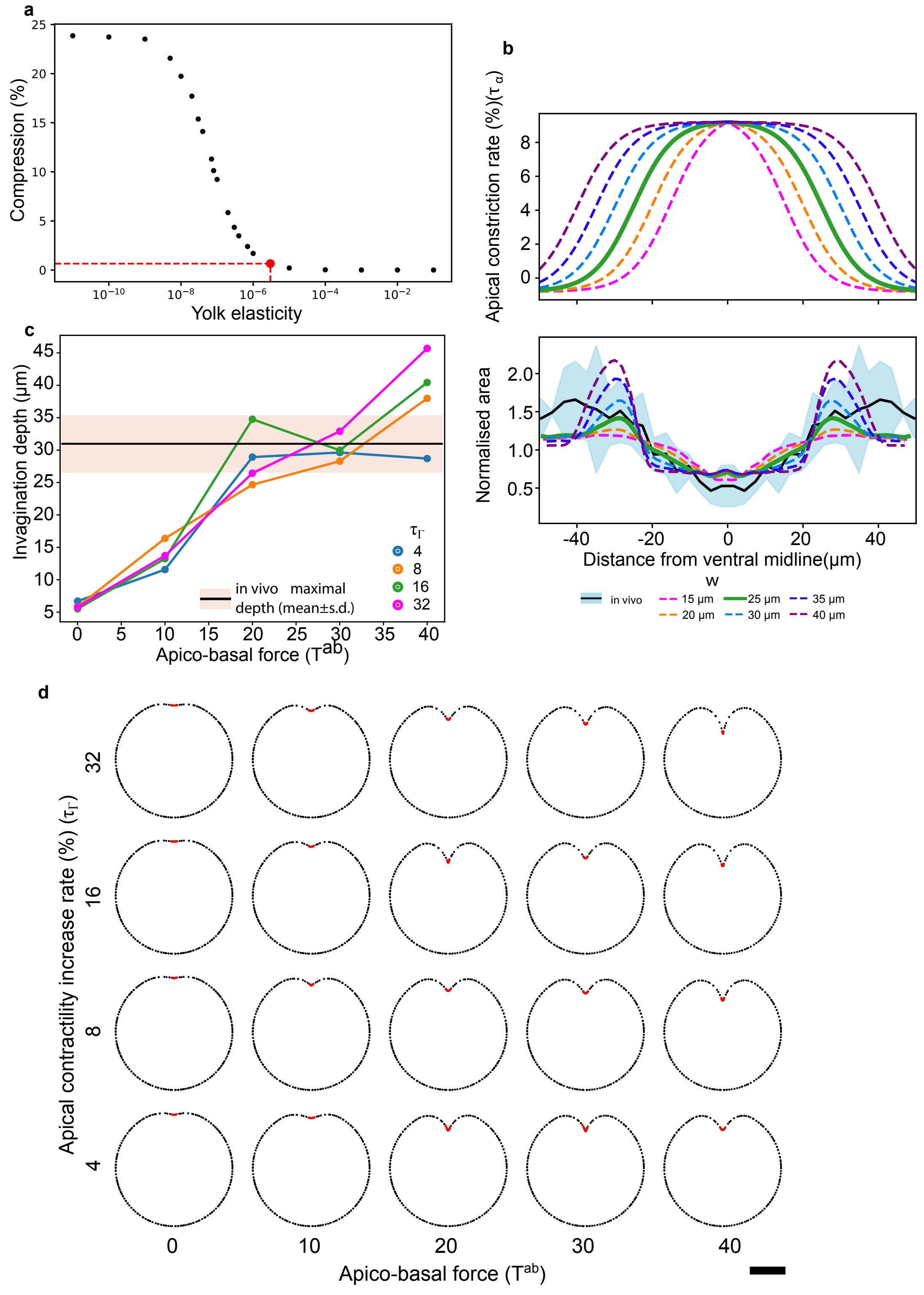

Supplementary Figure 4. Apico-basal traction is required for mesoderm invagination in a 3D biophysical model: Influence of parameter values on the model (Related to Figure 7, 8).

a. Yolk compression (V_γ/V_0) as a function of yolk volume elasticity (K_γ). The value of (K_γ) was chosen at the lowest value such that compression of the ellipsoid by cellular contractility was less than 1% in volume (red dot).

b. Top: Apical constriction rate plotted with respect to the distance from the ventral midline for different values of the profile width w (in μm) for τ_Γ of 8%. Bottom: resulting area distribution profiles of mesodermal cells at t0 in silico (colored lines) and in vivo (mean: black line, s.d.: bleu band, n=3 embryos). The continuous green line corresponds to the value of w chosen for the simulation of full invagination. No apico-basal force was exerted in these simulations.

c. Maximal invagination depth for different values of apico-basal force (T^ab in u/μm, abscissa) and apical contractility increase rate (each color denotes one value of τ_Γ in %). The black line with a pink border denotes the average depth measured in vivo at the end of invagination.

d. Transversal sections of the model embryo at full invagination for distinct values of contractility increase rate τ_Γ (y-axis) and apico-basal tension T^AB (x-axis). All other parameter values are unchanged. Black and red dots symbolize ectodermal and mesodermal cells respectively, and the grey line represents the initial shape of the embryo. All simulation snapshots were taken at the time step of maximal invagination depth. Scale bar: 50μm

See also Supplementary Movie 9.

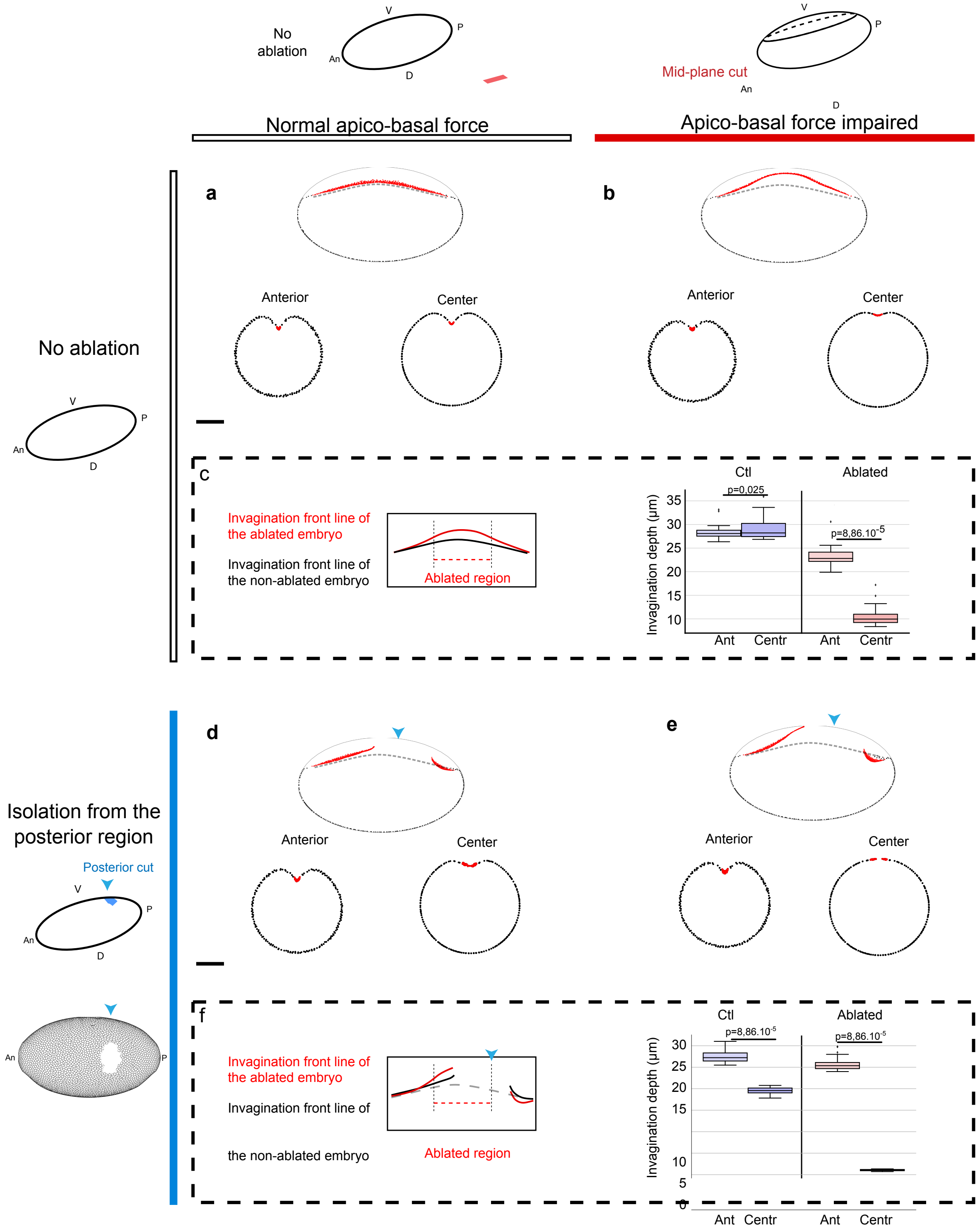

Supplementary Figure 5. Simulating laser ablations in a 3D biophysical model. (Related to Figure 6, Supplementary Fig 3)
Schemes recapitulate the different simulated ablations on top and on the left. The apical ablated region is indicated by blue arrowheads. The result can be seen on the ventral view of the simulated embryo. The mid-plane ablation (performed in b and e) is represented by a red rectangle in the scheme. Longitudinal and transversal views correspond to time step 50, at this time the invagination is considered as complete; the grey lines represent the initial shape of the embryo. Ectodermal cell edges are in black and mesodermal cells in red. An: anterior, P: posterior, V: ventral, D: dorsal The dashed grey lines in the transversal sections represent the position of the full invagination profiles for the control embryo (shown in a).
a-b. Longitudinal (top) and transversal (bottom) sections of the simulated embryos. Transversal sections from the central and the anterior region are presented below. The invagination after mid-plane ablation is perturbed in the central region (b, n=20) compared to the control condition (a, n=20). Scale bar: 50µm.
c. Left: Invagination front lines of the simulated embryos shown in a,b, highlighting the invagination defect in the central region compared to the anterior region in the ablated simulated embryo (red line) compared to the control (black line). Right: Invagination depth of the central region (Centr) compared to the anterior region (Ant) in control (ctl) and ablated simulated embryos. Statistical significance was assessed by the Wilcoxon signed-rank test (ctl: n=20, p=0,025; ablated: n=20, p= 〖8,86.10〗 ^(-5)).
d-e. Longitudinal (top) and transversal (bottom) sections of the simulated embryos. Transversal sections from the central and the anterior region are presented below. The invagination front is slightly curved in the control condition (d, n=20), whereas it is strongly curved and asymmetric when apico-basal forces are disrupted in the central region (e, n=20). Scale bar: 50µm.
f. Left: Invagination front lines of the simulated embryos in B, B' highlighting the curved invagination profile in ablated condition compared to the control. Right: Invagination depth of the central region with (ctl) or without (ablated) apico-basal force (see Methods), measured respectively at t0 and at the moment where the invagination is maximal. Statistical significance was assessed by the Mann-Whitney test (ctl: n=20 and ablated: n=20; at t0: p= 〖8,86.10〗 ^(-5) – significant; at the end of the invagination: p= 〖8,86.10〗 ^(-5) – significant).
