# Supplementary material for: Mechanical impact of epithelial−mesenchymal transition on epithelial morphogenesis in Drosophila
Source: Nat Commun. 2019 Jul 4;10:2951. doi: 10.1038/s41467-019-10720-0 (PMC6609679; doi:10.1038/s41467-019-10720-0)
Supplement: Supplementary file 12 — Reporting Summary [file 41467_2019_10720_MOESM12_ESM.pdf]

nature research

Corresponding author(s): Magali Suzanne Corinne Benassayag and Guillaume Gay

Last updated by author(s): Apr 26, 2019

Reporting Summary

Nature Research wishes to improve the reproducibility of the work that we publish. This form provides structure for consistency and transparency in reporting. For further information on Nature Research policies, see Authors & Referees and the Editorial Policy Checklist.

Statistics

For all statistical analyses, confirm that the following items are present in the figure legend, table legend, main text, or Methods section.

n/a	Confirmed	
☐	☒	The exact sample size (*n*) for each experimental group/condition, given as a discrete number and unit of measurement
☐	☒	A statement on whether measurements were taken from distinct samples or whether the same sample was measured repeatedly
☐	☒	The statistical test(s) used AND whether they are one- or two-sided<br>*Only common tests should be described solely by name; describe more complex techniques in the Methods section.*
☐	☒	A description of all covariates tested
☐	☒	A description of any assumptions or corrections, such as tests of normality and adjustment for multiple comparisons
☒	☐	A full description of the statistical parameters including central tendency (e.g. means) or other basic estimates (e.g. regression coefficient) AND variation (e.g. standard deviation) or associated estimates of uncertainty (e.g. confidence intervals)
☐	☒	For null hypothesis testing, the test statistic (e.g. *F*, *t*, *r*) with confidence intervals, effect sizes, degrees of freedom and *P* value noted<br>*Give P values as exact values whenever suitable.*
☒	☐	For Bayesian analysis, information on the choice of priors and Markov chain Monte Carlo settings
☒	☐	For hierarchical and complex designs, identification of the appropriate level for tests and full reporting of outcomes
☒	☐	Estimates of effect sizes (e.g. Cohen's *d*, Pearson's *r*), indicating how they were calculated

Our web collection on statistics for biologists contains articles on many of the points above.

Software and code

Policy information about availability of computer code

Data collection	Images were acquired using Zen black or Zen blue softwares on LSM710, LSM880 and Zeiss spinning disc; on LAS and metamorph on SP8 and Leica spinning disc.
Data analysis	We used Image J for image analysis (doi:10.1038/nmeth.2019), Matplotlib (https://dx.doi.org/10.5281/zenodo.1202077) and Ipyvolume libraries (https://dx.doi.org/10.5281/zenodo.1286976) for modelling results.

For manuscripts utilizing custom algorithms or software that are central to the research but not yet described in published literature, software must be made available to editors/reviewers. We strongly encourage code deposition in a community repository (e.g. GitHub). See the Nature Research guidelines for submitting code & software for further information.

Data

Policy information about availability of data

All manuscripts must include a data availability statement. This statement should provide the following information, where applicable:

- Accession codes, unique identifiers, or web links for publicly available datasets
- A list of figures that have associated raw data
- A description of any restrictions on data availability

Data availability
Results obtained from the vertex model are displayed using the Matplotlib (https://dx.doi.org/10.5281/zenodo.1202077) and Ipyvolume libraries (https://dx.doi.org/10.5281/zenodo.1286976). The data that support all experimental findings of this study are available from the corresponding authors upon reasonable request.

Field-specific reporting

Please select the one below that is the best fit for your research. If you are not sure, read the appropriate sections before making your selection.

☒ Life sciences ☐ Behavioural & social sciences ☐ Ecological, evolutionary & environmental sciences

For a reference copy of the document with all sections, see nature.com/documents/nr-reporting-summary-flat.pdf

Life sciences study design

All studies must disclose on these points even when the disclosure is negative.

Sample size	No sample size calculation was performed. Since there is no ethical obligation regarding the use of Drosophila melanogaster, sample size was limited on one hand by the time required to perform the experiment and on the other hand by the aim to have an accurate statistical inference.
Data exclusions	No data were excluded from the analysis.
Replication	All attemps of replication were successful. The phenotypes observed were highly reproducible as confirmed by the significance of the p values obtained in statistical tests.
Randomization	This is not relevant for this study since it relies on the description of a cellular dynamics coupled with microdissections experiments which can not be randomized. Control samples were always treated in the exact same condition as the micromanipulated ones (stage, culture, time frame of observation, time of sample illumination).
Blinding	This is not relevant for this study since it relies on the description of a cellular dynamics coupled with microdissections experiments which can not be blinded.

Reporting for specific materials, systems and methods

We require information from authors about some types of materials, experimental systems and methods used in many studies. Here, indicate whether each material, system or method listed is relevant to your study. If you are not sure if a list item applies to your research, read the appropriate section before selecting a response.

Materials & experimental systems

n/a	Involved in the study	
☐	☒	Antibodies
☒	☐	Eukaryotic cell lines
☒	☐	Palaeontology
☐	☒	Animals and other organisms
☒	☐	Human research participants
☒	☐	Clinical data

Methods

n/a	Involved in the study	
☒	☐	ChIP-seq
☒	☐	Flow cytometry
☒	☐	MRI-based neuroimaging

Antibodies

Antibodies used	Primary antibodies obtained from Developmental Studies Hybridoma Bank were: rat anti-E-Cad (DCAD2, 1/50) and rat anti-α-Catenin (DCAT-1, 1/50). Rabbit anti-Snail antibody was a gift from Leptin. Secondary antibodies coupled to Alexa-488, -555 and -647 were obtained from Fisher Scientific and diluted 1/200.
Validation	Primaries antibodies were previously described:<br>Anti-DE-cadh : https://www.citeab.com/antibodies/150741-dcad2-shg-antibody-dcad2<br>anti-α-Catenin: https://www.citeab.com/antibodies/150121-dcat-1-alpha-cat-antibody-dcat-1<br>anti-Snail :http://dev.biologists.org/content/develop/120/5/1137.full.pdf

Animals and other organisms

Policy information about studies involving animals; ARRIVE guidelines recommended for reporting animal research

Laboratory animals	Drosophila melanogaster were used in this study.
Wild animals	This study do not involved wild animals.
Field-collected samples	N/A

Ethics oversight

No ethical approval is needed to do research on Drosophila melanogaster.

Note that full information on the approval of the study protocol must also be provided in the manuscript.
